# Sleep Difficulties in Swiss Elite Athletes

**DOI:** 10.3390/life14060779

**Published:** 2024-06-20

**Authors:** Albrecht P. A. Vorster, Daniel Erlacher, Daniel Birrer, Philipp Röthlin

**Affiliations:** 1 Department of Neurology, University Hospital (Inselspital), University of Bern, 3010 Bern, Switzerland; 2 Interdisciplinary Sleep-Wake-Epilepsy-Center, University Hospital (Inselspital), University of Bern, 3010 Bern, Switzerland; 3 Institute of Sport Science, University of Bern, 3012 Bern, Switzerland; daniel.erlacher@unibe.ch (D.E.); philipp.roethlin@baspo.admin.ch (P.R.); 4 Elite Sport Department, Swiss Federal Institute of Sport Magglingen, 2532 Magglingen, Switzerland; daniel.birrer@baspo.admin.ch

**Keywords:** sleep, mental health, elite athlete, prevalence, sleep disorders

## Abstract

For athletes, sleep is essential for recovery and performance. Yet, up to two-thirds of athletes report poor sleep quality. Comprehensive data across all sports disciplines on the underlying causes of sleep problems are missing. We reanalyzed a data set of *N* = 1004 Swiss top athletes across an extensive array of 88 sports to gain knowledge on the specific deficits in sleep health with respect to gender, sport classes, sport-related factors, and well-being. We found that 18% of athletes were affected by at least two out of five high-risk sleep factors: 9% of athletes slept less than 6 h per day, 30% were dissatisfied with their sleep, 17% showed problems falling asleep within 30 min, 18% of athletes reported difficulty maintaining sleep more than three times a week, and 6% of athletes used sleeping pills more than once a week. We found sleep health strongly linked to overall well-being and mental health (22% showed at least moderate symptoms of either depression or anxiety). Therefore, screening and treating sleep disorders might effectively improve mental health and general well-being as well as performance among athletes around the globe.

## 1. Introduction

Sleep plays a pivotal role in recovery, optimal athletic performance, and overall health [1,2]. Specifically, sleep is essential to make accurate and fast decisions as well as for efficient movement skills during the game [3,4]. Poor or reduced sleep impedes mood and motivation [5], increases the injury frequency due to a lack of regeneration, and might reduce muscle glycogen, while increasing stress perception and reducing sprint performance [6,7].

Yet sleep problems among athletes appear to be as frequent as in the normal population, even though athletes are active, without chronic diseases, and typically non-obese [8,9]. Up to two-thirds of athletes report poor sleep quality [8,10,11]. Surveys of Swiss participants in the Olympic Games persistently found that between 20–34% of athletes suffer from some sort of sleep problems during the Olympics, negatively impacting their subjective performance assessment during the games [12]. Similar studies show even higher figures, with almost half of Australian Olympic athletes (49%) suffering from a lack of sleep [13]. Still, the underlying causes of sleep problems among athletes remain largely unknown. So far, organic causes for sleep problems have been largely neglected, despite several studies indicating that athletes appear to be surprisingly often affected by obstructive sleep apnea or restless legs syndrome [9]. Athletes also contend with environments that can disrupt sleep: these include the challenges of travel [14], competition stress [15], irregular bedtimes [16], frequent change of sleeping places due to competitions [17], and unfavorable training schedules [18]. Athletes usually must integrate school and work shifts into their schedules as only a tiny fraction of professional athletes are full-time employed and paid by their sport. Thus, they have to face academic and athletic requirements at the same time (e.g., [19]). Furthermore, high training loads may increase sleep problems [20]. Impaired sleep is a known indicator of non-functional overreaching and overtraining [21,22].

In order to fit with the unique sleep challenges faced by elite athletes due to their specific environment of training, competition schedules, and frequent travel, Samuels et al. [23] created the Athlete Sleep Screening Questionnaire (ASSQ). The ASSQ has been shown to be a quantitative reliable, athlete-specific clinical sleep-screening tool, that assesses the quality, length, and consistency of sleep and provides a global Sleep Difficulty Score (SDS). The ASSQ comprises 15 items focusing on key sleep domains: Total Sleep Time, Insomnia, Sleep Quality, and Chronotype, with additional considerations for modifiers like Travel Disturbance and Sleep Disordered Breathing. Five Likert-scale questions are summed to generate a SDS. A higher SDS indicates a greater likelihood of a clinical sleep problem placing athletes into one of four distinct categories indicating the severity of their sleep issues: none, mild, moderate, or severe. As there is a great need for suitable interventions, the ASSQ provides interventions based on SDS scores and modifiers.

In the original study, out of 349 athletes screened, 13.2% were identified as needing further consultation, with follow-up confirming the need for interventions in those flagged by the ASSQ. A subsequent study by Bender et al. (2018) [24] aimed to clinically validate the ASSQ with a five-item version involving 199 Canadian National Team athletes; the study assessed the ASSQ’s clinical validity through comparison with sleep medicine physician (SMP) evaluations. The ASSQ demonstrated good agreement with SMP assessments, exhibiting a diagnostic sensitivity of 81% and specificity of 93%. The study found that 25.1% of athletes required further sleep assessment, significantly lower than rates suggested by general sleep questionnaires unvalidated for athletes.

Lately, the translated ASSQ has been shown to be effective in screening sleep problems internationally. Zhang et al. (2022) [25] investigated sleep disturbances among Chinese athletes using the ASSQ. In total, 394 athletes aged 18–32 years (47.6% female) were assessed for theSDS, sleep quality, and various factors associated with sleep disturbances. They found 14.2% of athletes experiencing moderate to severe sleep problems, with travel and evening chronotype being significant contributors to poor sleep. A study by Rabin et al. (2020) [26] assessed sleep health in college athletes also by the ASSQ. With a sample of 1055 athletes from four NCAA institutions, it found that approximately 25% experienced clinically meaningful sleep problems. Higher college years were linked to worse sleep, highlighting a need for sleep health interventions in this population. With respect to the aforementioned studies, the ASSQ proved efficient for large-scale sleep health evaluation. These studies collectively underscore the ASSQ’s critical role in identifying and addressing sleep issues in athletes and its consistency across diverse athlete groups.

Currently, studies on the prevalence of sleep difficulties among elite athletes, particularly in large samples, are scarce. Specifically, comprehensive data for Swiss athletes are missing. In the present research, we seek to explore the specific sleep difficulties depending on sport classes and their relation to mental health. For that purpose, we delved deeper into a data set from a cross-sectional study, with a single measurement time point, previously featured in a publication by Röthlin et al. (2023) [27]. An online survey was sent out to all Swiss athletes that were either national squad members at an elite level or at the junior level, of which more than 20% participated.

This publication stands out as one of the first studies to evaluate sleep issues in a large cohort of 1004 elite athletes spanning all key sports categories across various ages and genders utilizing the ASSQ. This broad data set allowed us to identify target groups in which sleep problems are more prevalent than others, potentially pointing to underlying causes and points of action. Furthermore, to our knowledge, this is the first study using the ASSQ in combination with mental health questionnaires, namely the PHQ-9 and GAD-7, assessing the mental health of the athletes. This offered novel insights into the interplay between sleep difficulties, sport-related factors, and mental health across an extensive array of 88 sports.

## 2. Materials and Methods

### 2.1. Study Design

This cross-sectional study, with a single measurement time point, was based on a data set reported earlier [27].

### 2.2. Participants and Procedure

In brief, all Swiss athletes aged 16 years or older who fell into one of two national support categories were invited to participate in an online survey (*N* = 4872, *M_age_* = 22.4, *SD_age_* = 7.2). Athletes were either national squad members at an elite level or at the junior level—by definition, individuals who receive these supports are Switzerland’s best athletes in their respective sports. Each participant received a personal code and a link to the online survey. Each participant gave informed consent before commencing the online survey in their chosen language. This study was approved by the cantonal ethics committee (Project ID 2021-802282).

In total, 1004 athletes (*N* = 540, 53.8% female and *N* = 464, 46.2% male) completed the survey and were included in the analysis. The response rate was 21% of those contacted. Participants from 88 different sports completed the survey, the most common being soccer (6%), athletics (6%), ice hockey (6%), alpine skiing (6%), floorball (4%), rowing (3%), volleyball (3%), and orienteering (2%). To group the athletes from different sports, the participants were divided into eight sports groups according to a classification system used by the Norwegian Olympic Training Centre [28] (see Table 1): technical (e.g., curling, shooting), endurance (e.g., road cycling), aesthetic (e.g., synchronized swimming), weight-class (e.g., wrestling), ball game (e.g., soccer), power (e.g., ski alpine), antigravitation (e.g., climbing), and diverse sports (e.g., pentathlon). On average, they had 14.8 (±6.5) weekly training hours and 26.7 (±18.5) competitions per year.

### 2.3. Measures

Sleep was assessed via the 5 items for the Sleep Difficulty Score (SDS) from the Athlete Sleep Screening Questionnaire (ASSQ, see Table 2) [23,24]: (1) sleep duration, (2) sleep satisfaction, (3) sleep latency, (4) sleep maintenance, and (5) the use of sleep medication. Item 1 was rated on a 5-point Likert scale ranging from 5 to 6 h (4) to more than 9 h (0), item 2 on a Likert scale ranging from very satisfied (0) to very dissatisfied (4), item 3 on a 4-point Likert scale ranging from 15 min or less (0) to longer than 60 min (3), item 4 on a 4-point Likert scale ranging from none (0) to five to seven days per week (3), and item 5 on a 4-point Likert scale ranging from none (0) to five to seven times per week (3). By adding up the scores from all five items, a cumulative score between 0 and 17 is achieved. Cut-points were suggested to categorize athletes into clinical sleep problem of none (0–4), mild (5–7), moderate (8–10), and severe (11–17). This scoring method has been shown to have a sensitivity of 0.81 and a specificity of 0.93 [24]. Whereas in the original study the SDS showed acceptable internal consistency with a Cronbach’s alpha value of 0.74, in our study group, the SDS demonstrated only an internal consistency with a Cronbach’s alpha value of 0.50—this suggests that the items within the instrument are not coherently measuring the same underlying construct of sleep difficulties which has implications for the statistical analysis (see below). The original questions posed in German and French are provided in the Table S1.

Unfortunately, the SDS applies a weighting where no problematic behavior might be present. For instance, additional difficulty score points are assigned for every hour sleeping less than 9 h of sleep duration or falling asleep in more than 15 min. Both behaviors fall in the normal, healthy range and thus should not receive additional difficulty points. Therefore, we converted the items of the five sleep factors of the ASSQ with respect to the concept of a sleep health score [29], assigning one point for good sleep behavior and 0 points for poor sleep behavior in analogy to Fan et al. (2019) [30] (Table 2). High-risk sleep factors were defined as follows: (1) sleep less than 6 h per day, (2) sleep dissatisfaction, (3) sleep latency of more than 30 min, (4) sleep maintenance problems of more than twice per week, and (5) sleep medication use. For each sleep factor, athletes received a score of 1, whether they were classified as high risk for that factor or 0 if at low risk for that factor.

In the original study focusing on mental health in athletes from Röthlin et al. (2023) [27], relevant health factors were reported that are relevant to sleep disturbance. First, depressive symptoms in the two preceding weeks prior to assessment were measured via the 9-item Patient Health Questionnaire (PHQ-9) [31,32,33]. Cut-off points of 5, 10, and 15 are classified as mild, moderate, and severe depression, respectively. Second, symptoms of anxiety in the two preceding weeks were assessed via the 7-item General Anxiety Disorder (GAD-7) [34,35,36]. Cut-off points of 5, 10, and 15 are classified as mild, moderate, and severe anxiety, respectively. Prevalence was determined by the percentage of participants who met or exceeded specific threshold levels for symptoms of mental disorders [37]. These threshold levels were set at 10 for the PHQ-9 and GAD-7 assessments, and at 8 for the ASSQ. And third, well-being was assessed via the 14-item Mental Health Continuum Short Form (MHC-SF) [38,39,40]. For this sample, the MHC-SF achieved the following internal consistency values: emotional well-being α = 0.86; social well-being α = 0.80; psychological well-being α = 0.84.

Finally, the survey included several questions that were sport-related: (1) weekly training hours, (2) the number of competitions, and (3) injury that prevented training for at least another 2 weeks (1 = yes, 0 = no).

### 2.4. Statistics

As studies on the prevalence of sleep difficulties in large samples of elite athletes are scarce, the focus of this study is mainly on descriptive analysis.

Regression analysis with the ASSQ as the dependent variable was calculated to investigate possible influencing factors of sport type (included as dummy variables for all 8 sports categories), weekly training hours, number of competitions, and injuries. As gender and age are related to sleep difficulties and might be possible confounders, those variables were included in the regression as control variables. Due to the low internal consistency of theSDS, we applied ordinal regressions to all five ASSQ items. Furthermore, Röthlin et al. (2023) [27] have shown that depressive symptoms, anxiety symptoms, and general well-being have high correlations to each other, and to theSDS, so we decided to only include general well-being in the regression analysis.

Statistical analyses were performed with IBM SPSS Statistics for Windows (version 29.0)

## 3. Results

**Figure 1:**
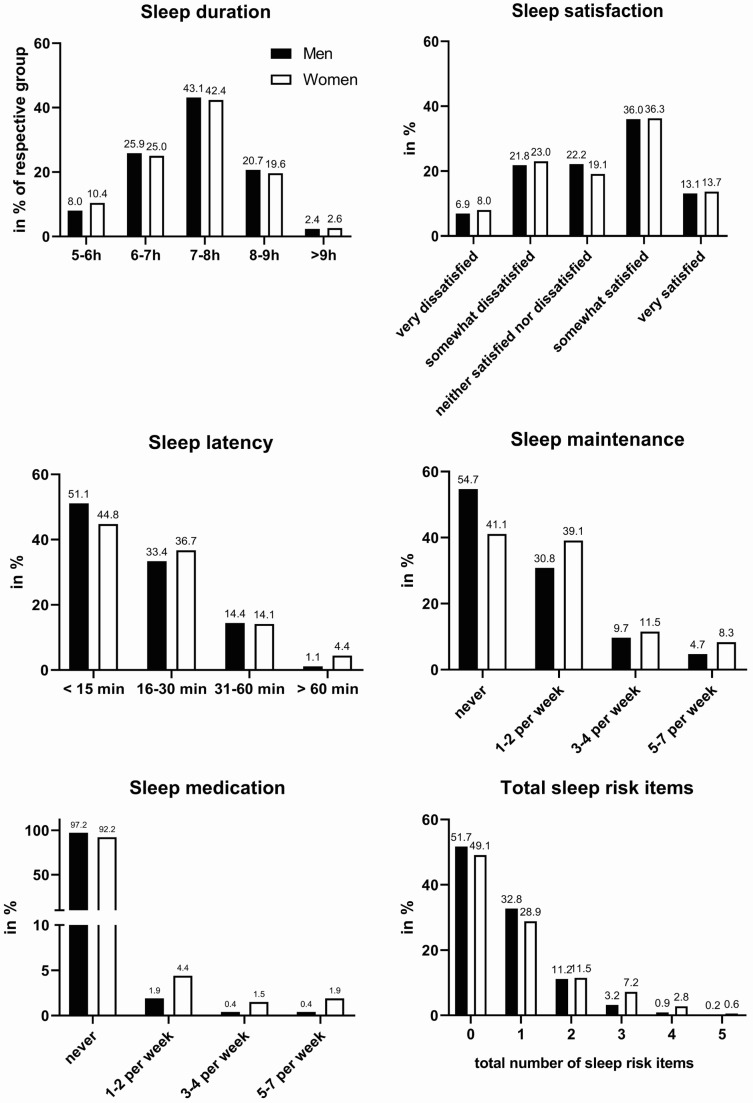
Sleep domains of the ASSQ in Swiss elite athletes. Answers are shown in percent of the respective group (men = black bars, women = white bars).

First, we will report the data for the five items of the ASSQ (Figure 1). Athletes slept on average 7–8 h, with no difference between men and women. Sleep satisfaction emerged as a significant issue, with 30% of athletes somewhat or very dissatisfied with their sleep, including 8% very dissatisfied. Again, there was no difference between men and women. Across all sports, 17.3% of the athletes sampled showed problems falling asleep within 30 min, meeting a key criterion for insomnia, with approximately 3% facing severe onset problems (over 60 min). Women were more prone to facing challenges in falling asleep (*p* < 0.05). In total, 18% of athletes reported difficulty maintaining sleep more than three times a week, thus fulfilling another medical core criterion for insomnia. Again, women were more likely to encounter problems maintaining sleep through the night (*p* > 0.001). Sleep medication consumption was high among athletes, with 5.5% of athletes using sleeping pills more than once a week. Females were more than twice as likely to take sleeping aids (7.8% of women vs. 2.7% of men; *p* > 0.001). When the ASSQ scoring system was applied as a SDS to the entire sample, 19.0% of athletes were identified as having a moderate or severe level of clinical sleep problem (None: 37.4%, Mild: 43.6%, Moderate: 15.1%, and Severe: 3.9%).

Second, we report the percentage of the risks for each of the five items mentioned above for the different sports categories, team and individual sports as well as for different age groups (see Table 3). Sleep duration was increased in individual sports compared to team sports. Sleep duration was also longer in endurance, power, and antigravity than in other sports. Interestingly, athletes under 18 years of age slept less than older athletes between 19 and 25 years of age. Sleep duration decreases with age, as seen in athletes over 40 years old. We did not find any differences between sports groups, team/individual sports, or timing of sports (morning or evening sports groups). We found no differences between team or individual sports. It appeared as if aesthetic sports are more prone to this type of sleep problem. However, the number of athletes in that respective group was rather small, and women were four times overrepresented in this group, which made a profound statement. Sleep dissatisfaction is similarly high across all sports. Satisfaction is highest in power sports. We found no difference in terms of gender or team/individual sports. In addition, we found a cultural difference: French-speaking athletes were almost twice as likely to take sleeping pills as German-speaking athletes (8.4% vs. 4.7%; not depicted in the table). We found that 19% of athletes had a sleep health score of 3 or lower. This means they had sleep problems in at least two domains. In total, 7.7% of the athletes had sleep problems in at least three areas. Their sleep was severely disturbed and they may need treatment. Women were more than twice as likely to have such combined sleep problems.

Third, we report the results of the regression analyses. Sleep is closely related to depression and anxiety disorders (see Table 4). Not surprisingly, we found that both disorders were strongly correlated with each of the sleep items—here, listed in order of importance: 1. subjective sleep duration, 2. problems of sleep maintenance (sleep efficiency), 3. problems initiating sleep (sleep latency), 4. use of sleep medication, and 5. satisfaction with sleep quality.

In our population, 17% of athletes reported moderate to severe symptoms of depression in the past two weeks, while 10% of the athletes reported moderate to severe symptoms of anxiety. In total, 22,4% of athletes had at least moderate symptoms of either depression or anxiety, i.e., scoring above the cut score of 10 on the PHQ-9 or GAD-7. Of these, more than half expressed at least one clear sign of insomnia (problems falling asleep within 30 min or problems more than three times a week maintaining sleep or taking sleep medication more than once a week).

Overall well-being (assessed via the 14-item Mental Health Continuum Short Form) was most strongly impacted by symptoms of depression or anxiety, both of which were affected in turn by the different sleep domains (Figure 1).

Surprisingly, we found that sleep problems decreased with the number of training hours. Sports with a body weight target (leanness) did not tend to have lower sleep health than other sports groups. Finally, we did not find a correlation between the sleep health score and whether the athlete suffered from an injury at the time of participation in the online survey.

## 4. Discussion

We examined sleep disturbances and their connection to other mental health and sport-related variables for the first time in a large representative sample of Swiss elite athletes. We found that 19% of athletes showed a moderate or severe level of clinical sleep problems measured by the ASSQ. Furthermore, 7.7% of the athletes showed sleep problems in at least three domains, pointing to a sleep behavior where professional advice is recommended. Our study is one of the first representative national samples in the world covering all major sports disciplines, showing that every fifth professional athlete expresses sleep deficits in a combination of essential domains of sleep health and thus does not merely suffer from insufficient sleep. Our work is in line with previous work from other countries using the same instrument, demonstrating that 25% of Canadian National team athletes [24], 25% of US American collegiate athletes [26], and 14% of Chinese national-level athletes [25] exceeded the cut-off for moderate sleep problems in the ASSQ score and thus show a sleep behavior that indicates room for improvement. Thus, it appears to be a global phenomenon that a substantial proportion of elite athletes (14–25%) suffer from sleep problems, a largely untouched reservoir to improve performance.

We found, as did others, that women report sleep problems more often than men [41]. However, women tend to report sleep problems more often than men [42] while objectively showing better sleep quality than men [43]. Yet this finding might be merely due to a reporting bias.

In our data set, we investigated, in particular, sleep duration, sleep satisfaction, sleep latency and efficiency, the use of sleep medication, as well as symptoms of depression, anxiety, and well-being. For sleep duration, we found that across all sports classes, 35% of athletes slept up to 7 h during the night. However, most athletes need about 8 h of sleep per night to feel rested [44]. While we did not find a gender difference in sleep duration, we found that athletes of individual sports tended to sleep longer than members of team sports. This contrasts with Sargent et al. 2021 [38], who found that athletes involved in team sports obtained more sleep (6.9 h) than athletes involved in individual sports (6.4 h). Yet our sample was larger and spanned more disciplines. One possible explanation for our finding is that training schedules in teams are rather fixed, while single-sport athletes have the chance to negotiate training times with their trainers. In addition, most team sports (especially ball games) favor evening competition events. Frequent evening competitions and training tend to delay bedtime and thus reduce the sleep window. This idea is nevertheless not supported by our regression analysis, even though there is a tendency that in endurance, power, and antigravity disciplines, longer sleep durations were reported. Again, this result might be due to the fact that these sports classes tend to be rather single disciplines than team sports, leading to the same causes as described above.

We found a shortened sleep duration in adolescent athletes (<18 years) compared to athletes ranging from 19–25 years, pointing to a systematical sleep deficit in this younger age group. In this group of young athletes, 35% fail to meet the recommended sleep duration of eight to ten hours [45] by the American Academy of Sleep Medicine (AASM), similar to what is found in the normal population [43]. Daytime sleepiness is most prevalent in teenagers, as 18% of this group reports daytime sleepiness. Shortened sleep during adolescence is mainly caused by a 3–5 h delayed secretion of melatonin. As a result, the ability to fall asleep is shifted by 3–5 h starting in puberty, while school times and rise times remain early. Late training schedules, in combination with early school starts, might leave too little time for sleep. Daytime sleepiness is usually a result of insufficient sleep or disturbed sleep, like sleep apnea [43]. Thus, allowing young athletes to sleep sufficiently might yield the biggest impact on improving their sleep health. It is already known that only a minority of 3% of athletes tend to get the amount of sleep that they would subjectively need to feel refreshed [44]. It is currently being discussed whether late training does more harm than good, as a higher training load has been shown to reduce sleep duration. Actual sleep duration may differ depending on whether competitive sports are played. Overall, the sleep deficit in athletes might be greater than in the general public as athletes tend to require one hour of additional sleep for recovery as a rule of thumb [46] while showing an even shorter sleep duration [47].

Advising athletes to go to bed earlier might not always be helpful advice: 17% of our sample struggle habitually to fall asleep within 30 min, indicating that they potentially go to bed too early according to their chronotype. Alternatively, these athletes might not have established a calming sleep routine or might be too aroused by regular evening training or matches [9,42]. We have to note that daytime sleep (napping) was not included in this survey. Thus, it is possible that some of the athletes already compensate for the missed night-time sleep by daytime napping.

In addition, 18% of athletes have difficulty staying asleep throughout the night more than three times a week. Female athletes are more frequently affected by problems of sleep maintenance, just like in the general population. One potential explanation is that women are more likely to show certain personality factors that favor insomnia, such as neuroticism and agreeableness (altruism, empathy, considerateness) [48,49]. In response to stress, women appear to be more likely to respond with sleep problems, termed “stress reactivity” [50]. In line with this reasoning, we also found that athletes in aesthetic sports are more prone to problems maintaining sleep, which could potentially be due to the personality traits of perfection in these sports.

The surprisingly high rate of sleep medication use in our sample suggests that at least 5% of elite athletes regularly have severe problems initiating or maintaining sleep. As in the general population, women are more likely to take sleeping pills [43]. We found that female athletes were three times as likely to take sleeping pills more than three times per week (3.4% of females vs. 0.8% of males). The high rates in our sample match the rates found in the general population age group between 26–40, that is, 8.6% for women and 5.2% for men taking sleep medication on a regular basis [43].

In total, we found that 30% of our cohort had at least one core sign of insomnia: that is, problems falling asleep within 30 min, problems maintaining sleep more than three times a week, or taking sleep medication more than once a week. Insomnia symptoms appear to be a general problem across all sports types, as we did not find any differences in terms of sports classes or whether sports were classified as team or individual sports.

Sleep is closely related to depression and anxiety disorders. Not surprisingly, we found that both disorders were strongly correlated with each of the sleep items—here, listed in order of importance: 1. subjective sleep duration, 2. problems of sleep maintenance (sleep efficiency), 3. problems initiating sleep (sleep latency), 4. use of sleep medication, and 5. satisfaction with sleep quality.

Insomnia is a known risk factor for mental disorders like depression and anxiety [51]. A total of 85% of patients with a depressive disorder show insomnia symptoms [52]. Importantly, impaired sleep during adolescence predicts mental health problems in later life [53,54]. In our sample, we found that both depression and anxiety were strongly correlated with each of the sleep items, foremost sleep duration. Participants under the age of 18 revealed the lowest sleep duration. Thus, targeting proper sleep duration and quality might prevent a certain amount of depressive and anxiety symptoms in athletes. This is of great need as, in our study, nearly one-quarter of athletes showed at least moderate symptoms of either depression or anxiety (17% moderate to severe symptoms of depression and 10% moderate to severe symptoms of anxiety). Of these, more than half expressed at least one clear sign of insomnia.

Our findings have the following implications on athlete health and performance: as there is a strong effect of sleep quality on athletic performance, we can conclude that the performance of at least 19% of the elite athletes can be considerably enhanced, as this proportion is lacking good sleep quality. Sleep thus stands out as one of the key deficits in athlete health and preparation. We recommend that sleep disorders should be routinely screened (for example, via the ASSQ) and treated by sports physicians. Screening and treating sleep disorders is an effective way of preventing and partially treating mental health disorders that are closely related to sleep health. We recommend coaches to analyze the sleep need and sleep habits of their athletes by using sleep protocols to adapt training times appropriately, as we have found that late training might reduce sleep duration. We found sleep duration most closely related to mood disorders. We would like to advise sports physicians to consider cognitive behavioral therapy for insomnia instead of sleeping pills as this is the gold standard treatment and most appropriate therapy for insomnia, lacking the negative side effects on performance as sleep medication. As sleep can be enhanced among athletes through online courses [16,55,56], this would be one important way to educate athletes on this important topic.

Finally, it should be stated that the current study has two further limitations that warrant acknowledgment. Firstly, the use of self-report measures introduces significant subjectivity and potential biases. Participants’ responses may be influenced by social desirability, recall inaccuracies, and a lack of objectivity, which can compromise the reliability and validity of the findings. To mitigate these issues, future studies should consider employing objective measures such as polysomnography (PSG) and actigraphy. PSG, the gold standard for sleep studies, provides comprehensive data on brain activity, eye movements, muscle activity, heart rhythm, and breathing, allowing for a more detailed and accurate assessment of sleep parameters. Actigraphy, with its wrist-worn devices, offers a practical means to monitor sleep patterns and duration over extended periods in naturalistic settings, bridging the gap between lab-based PSG and self-reported sleep data. Additionally, it is important to address the low Cronbach’s alpha observed for the SDSin this study, which indicates potential reliability issues. A low alpha suggests that the items on the SDSmay not be consistently measuring sleep difficulty, raising concerns about the validity of the results related to sleep problems. Investigating the reasons behind this low reliability could involve examining item inter-correlations and considering whether the scale is suitable for the study’s population or if it requires modifications. As previously discussed, unfortunately, the SDSapplies a weighting where no problematic behavior might be present and therefore cannot add to a single score. A solution is our approach to examine the items separately, which does not mean that in the future a consistent sleep difficulty measure for athletes will be developed.

Improving sleep quality in athletes is important as sleep disturbances negatively affect athletic performance and injury risk [57], as well as immune regulation. Athletes sleeping less than 8 h per night showed a 1.7% increased risk of injury. Conversely, athletes can reduce the risk of injury by 61% by adhering to sleep recommendations.

The present study has several limitations. First, the present study is a cross-sectional online study with a single measurement time point with self-reported measures. Thus, we do not know whether sleep difficulties expressed here were of persistent nature and whether athletes provided true information on their sleep quality. Therefore, this study did not use any objective measures such as actigraphy or PSG in order to confirm diagnosis in athletes with high risk of sleep disorders. Finally, we found that the SDSdemonstrated only an internal consistency with a Cronbach’s alpha value of 0.50—this suggested that the items within the instrument are not coherently measuring the same underlying construct of sleep difficulties. This had implications on our analysis.

Further research is needed on effective behavioral training programs of sleep health for athletes and coaches showing effective ways to implement good sleep into their training schedules.

Key Summary Points

- 18% of Swiss top athletes have poor sleep health, in line with data from other nations (Canada, USA) tested with the same instrument.
- Female athletes are more prone to sleep problems, reporting a longer duration of falling asleep, more frequent troubles maintaining sleep, and a doubled use of sleep medication.
- Poor sleep health is linked to inferior mental health. Self-reported sleep duration was most sensitively linked to depressive symptoms and symptoms of anxiety disorder.
- Screening and treating sleep disorders might effectively improve mental health, general well-being, as well as performance among athletes around the globe. Effective screening instruments have to be developed.

## Figures and Tables

**Table 1:** Overview of the sample of the 1004 Swiss elite athletes who completed the online survey.

Sports Group	All	Men	Women	Age
	*N* (%)	*n* (%)	*n* (%)	*M* ± *SD*
Technical (e.g., curling, shooting)	215 (21.4%)	94 (43.7%)	121 (56.3%)	25.1 ± 10.4
Endurance (e.g., road cycling)	146 (14.5%)	75 (51.4%)	71 (48.6%)	22.6 ± 7.6
Aesthetic (e.g., synchronized swimming)	31 (3.1%)	7 (22.6%)	24 (77.4%)	19.4 ± 3.3
Weight-class (e.g., wrestling)	89 (8.9%)	49 (55.1%)	40 (44.9%)	21.1 ± 4.9
Ball game (e.g., soccer, ice hockey)	360 (35.9%)	171 (47.5%)	189 (52.5%)	19.7 ± 4.6
Power (e.g., ski alpine)	74 (7.4%)	30 (40.5%)	44 (59.5%)	21.1 ± 4.1
Antigravitation (e.g., climbing)	20 (2.0%)	9 (45.0%)	11 (55.0%)	20.7 ± 3.7
Diverse (e.g., track athletics, pentathlon)	69 (6.9%)	29 (42.0%)	40 (58.0%)	21.3 ± 5.9
Team sport	380 (37.9%)	185 (39.9%)	195 (36.1%)	20.6 ± 5.7
Individual sport	624 (62.2%)	279 (60.1%)	345 (63.9%)	22.3 ± 7.7
16–17	328 (32.7%)	145 (31.2%)	183 (33.9%)	16.6 ± 0.5
18–25	487 (48.5%)	224 (48.3%)	263 (48.7%)	20.6 ± 2.3
26–40	161 (16.0%)	84 (18.1%)	77 (14.3%)	29.9 ± 3.7
41–65	28 (2.8%)	11 (2.4%)	17 (3.1%)	50.8 ± 6.0
Total	1004 (100%)	464 (46.2%)	540 (53.8%)	21.6 ± 7.0

**Table 2:** Items of the Athlete Sleep Screening Questionnaire (ASSQ) [23,24]. We grouped the response options into categories of high-risk sleep behavior or low-risk sleep behavior [29,30].

	Sleep Duration (ASSQ_v1)	Sleep Satisfaction (ASSQ_v2)	Sleep Latency (ASSQ_v3)	Sleep Maintenance (ASSQ_v4)	Sleep Medication (ASSQ_v5)
	During the recent past, how many hours of actual sleep did you get at night?	How satisfied/dissatisfied are you with the quality of your sleep?	During the recent past, how long has it usually taken you to fall asleep each night?	How often do you have trouble staying asleep?	During the recent past, how often have you taken medicine to help you sleep (prescribed or over-the-counter)?
High-risk sleep factors (1 pt)	(4) 5 to 6 h	(4) very dissatisfied (3) somewhat dissatisfied	(3) >than 60 min (2) 31 to 60 min	(3) 5 to 7 days/week (2) 3 or 4 times/week	(3) 5 to 7 times/week (2) 3 or 4 times/week (1) 1 or 2 times/week
Low-risk sleep factors (0 pt)	(3) 6 to 7 h (2) 7 to 8 h (1) 8 to 9 h (0) >9 h	(2) neither satisfied nor dissatisfied (1) somewhat satisfied (0) very satisfied	(1) 16 to 30 min (0) <15 min	(1) 1 or 2 times/week (0) none	(0) none

**Table 3:** High risk for single sleep items.

	*n*	Sleep Duration	Sleep Satisfaction	Sleep Latency	Sleep Efficiency	Sleep Medication
all	1004	9.3%	29.9%	17.1%	17.3%	5.5%
technical	215	12.6%	34.0%	16.7%	22.3%	7.9%
endurance	146	7.5%	30.8%	20.5%	18.5%	8.2%
aesthetic	31	12.9%	45.2%	19.4%	25.8%	12.9%
weight-class	89	10.1%	27.0%	12.4%	16.9%	4.5%
ball game	360	9.7%	29.7%	16.9%	13.6%	2.5%
power	74	2.7%	18.9%	14.9%	13.5%	5.4%
antigravitation	20	0%	25.0%	25.0%	5.0%	0%
diverse	69	7.2%	26.1%	17.4%	23.2%	7.2%
team	380	9.7%	27.6%	17.6%	13.9%	2.6%
individual	624	9.0%	31.3%	16.8%	19.4%	7.2%
16–17	328	9.5%	29.0%	19.5%	9.8%	4.3%
18–25	487	8.0%	29.0%	15.8%	19.1%	5.5%
26–40	161	9.9%	36.0%	17.4%	21.7%	6.2%
41–65	28	25.0%	21.4%	10.7%	50.0%	14.3%

**Table 4:** Regression analysis for the single items of the ASSQ.

Dependent Variables	Sleep Duration	Sleep Satisfaction	Sleep Latency	Sleep Efficiency	Sleep Medication
age	−0.037 **	0.003	−0.008	0.055 **	0.027
Gender (1 = m, 2 = f)	−0.007	−0.101	−0.102	−0.455 **	−0.966 **
Overall well-being	0.419 **	0.483 **	−0.479 **	−0.451 **	−0.606 **
Training hours (week)	0.037 **	−0.008	0.009	0.020	0.033
Competitions	0.009 *	−0.002	−0.003	0.001	0.004
Injuries (0 = *n*, 1 = y)	−0.072	−0.227	−0.202	−0.199	−1.078 **
Technical	0.547 *	0.241	0.006	0.355	0.121
Endurance	−0.541	0.059	−0.186	0.472	−0.011
Aesthetic	0.471	0.483	0.096	−0.274	0.289
Weight-class	0.474	0.211	0.332	0.410	0.801
Ball game	0.448	0.062	−0.084	0.372	1.271 *
Power	−0.467	−0.526	−0.256	0.488	1.059
Antigravitation	0.033	0.266	−0.542	1.241 *	16.373

Note. Figures shown are standard coefficients (i.e., beta values); *n* = 1004, * *p* < 0.05, ** *p* < 0.01.

## Data Availability

This study is part of a three-year research project on mental health in competitive sports. The data will be made available upon completion of the project (12/2024) in a form that ensures the anonymity of the participants under this link https://doi.org/10.17605/OSF.IO/8K2XJ (access on 1 January 2025).

## Supplementary Materials

The following supporting information can be downloaded at: https://www.mdpi.com/article/10.3390/life14060779/s1, Table S1: German and French translation of the ASSQ items used in the study.
