# Supplementary material for: Sleep Difficulties in Swiss Elite Athletes
Source: Life (Basel). 2024 Jun 20;14(6):779. doi: 10.3390/life14060779 (PMC11204926; doi:10.3390/life14060779)
Supplement: Supplementary file 1 [file life-14-00779-s001.zip › life-3050750-supplementary.pdf]

Supplemental Material

Domain	Question
**Sleep Duration** (ASSQ_v1)	1. "During the recent past, how many hours of actual sleep did you get at night?"<br>German:<br>Wie viele Stunden hast du in letzter Zeit nachts tatsächlich geschlafen?<br>• 0 5-6 Stunden<br>• 1 6-7 Stunden<br>• 2 7-8 Stunden<br>• 3 8-9 Stunden<br>• 4 mehr als 9 Stunden<br><br>French:
**Sleep Satisfaction** (ASSQ_v2)	2. "How satisfied/dissatisfied are you with the quality of your sleep?"<br>German:<br>Wie zufrieden/unzufrieden bist du mit der Qualität deines Schlafs?<br>• 0 sehr unzufrieden<br>• 1 eher unzufrieden<br>• 2 weder unzufrieden noch zufrieden<br>• 3 eher zufrieden<br>• 4 sehr zufrieden<br>French:
**Sleep Latency** (ASSQ_v3)	3."During the recent past, how long has it usually taken you to fall asleep each night?"<br>German:<br>Wie lange hast du in letzter Zeit gewöhnlich gebraucht, um einzuschlafen?<br>• 0 15min oder weniger<br>• 1 16-30 min<br>• 2 31-60 min<br>• 3 länger als 60 min<br>French:
**Sleep Efficiency** (ASSQ_v4)	4. "How often do you have trouble staying asleep?"<br>German:<br>Wie oft hast du Mühe die ganze Nacht durchzuschlafen?<br>• 0 nie<br>• 1 1-2 Mal pro Woche<br>• 2 3-4 Mal pro Woche<br>• 3 5-7 Mal pro Woche<br>French:
**Sleep Medication** (ASSQ_v5)	5. "During the recent past, how often have you taken medicine to help you sleep (prescribed or over-the-counter)?"<br>German:<br>Wie oft hast du in letzter Zeit Medikamente genommen, die dir beim Schlafen helfen (verschrieben oder rezeptfrei)?<br>• 0 nie<br>• 1 1-2 Mal pro Woche<br>• 2 3-4 Mal pro Woche<br>• 3 5-7 Mal pro Woche<br>French:

Supplemental Table S1: German and French translation of the ASSQ items used in the study.
